# Both anti-TNF and CTLA4 Ig treatments attenuate the disease severity of *staphylococcal dermatitis* in mice

**DOI:** 10.1371/journal.pone.0173492

**Published:** 2017-03-06

**Authors:** Manli Na, Wanzhong Wang, Ying Fei, Elisabet Josefsson, Abukar Ali, Tao Jin

**Affiliations:** 1 Department of Rheumatology and Inflammation Research, Institute of Medicine, Sahlgrenska Academy at Gothenburg University, S-41346 Göteborg, Sweden; 2 Department of Medical Biosciences, Umeå University, Umeå, Sweden; 3 Department of Microbiology and Immunology, The Affiliated Hospital of GuiZhou Medical University, Guiyang, P.R. China; 4 Department of Rheumatology, Sahlgrenska University Hospital, Göteborg, Sweden; Katholieke Universiteit Leuven Rega Institute for Medical Research, BELGIUM

## Abstract

**Background:**

RA patients being treated with biologics are known to have an increased risk of infections. We recently demonstrated that both CTLA4 Ig and anti-TNF treatment aggravate systemic *Staphylococcus aureus* (*S*. *aureus*) infection in mice, but with distinct clinical manifestations. However, the effects of CTLA4 Ig and anti-TNF treatments on a local *S*. *aureus* infection (e.g., skin infection) might differ from their effects on a systemic infection.

**Aims:**

The aim of this study was to examine the differential effects of anti-TNF versus CTLA4 Ig treatment on *S*. *aureus* skin infections in mice.

**Method:**

Abatacept (CTLA4 Ig), etanercept (anti-TNF treatment) or PBS was given to NMRI mice subcutaneously inoculated with *S*. *aureus* strain SH1000. The clinical signs of dermatitis, along with histopathological changes due to skin infection, were compared between the groups.

**Results:**

Both CTLA4 Ig and anti-TNF treatment resulted in less severe skin infections and smaller post-infectious hyperpigmentation compared with controls. Consistent with the clinical signs of dermatitis, smaller lesion size, more epithelial hyperplasia and more granulation were found in skin biopsies from mice receiving anti-TNF compared with PBS controls. However, both CTLA4 Ig and anti-TNF therapy tended to prolong the healing time, although this finding was not statistically significant. Serum MCP-1 levels were elevated in the anti-TNF group relative to the CTLA4 Ig and PBS groups, whereas IL-6 levels were higher in PBS controls than in the other two groups. Both anti-TNF and CTLA4 Ig treatments tended to down-regulate the necrosis/apoptosis ratio in the locally infected skin tissue. Importantly, no tangible difference was found in the bacterial burden among groups.

**Conclusion:**

Both CTLA4 Ig and anti-TNF therapies attenuate disease severity but may prolong the healing time required for *S*. *aureus* skin infections. Neither treatment has an impact on bacterial clearance in skin tissues.

## Introduction

The last few decades have seen the emergence of new drugs for rheumatoid arthritis (RA) and other autoimmune diseases. Some of these drugs are biologics, which target specific cells and molecules of the immune system. The development of biologics has revolutionized how RA patients are treated, and they have significantly improved the quality of life of many patients who were not responding to traditional disease-modifying anti-rheumatic drugs (DMARDs) [1].

Even before treatment of RA with biologics became standard, RA patients were at an increased risk of developing serious infections. RA patients had greater frequency of infections such as skin and soft tissue infections, as well as septic arthritis compared to the general population [2, 3]. TNF inhibitors are one of the primary types of biologics currently in use. Patients on TNF inhibitors are known to have an increased risk of granulomatous infectious diseases [4, 5], some viral infections [6] and reactivation of hepatitis [7]. The risk of infections in RA patients receiving anti-TNF therapy is higher during the first few months following initiation of treatment. Subsequently, the risk starts to decrease or remains stable the longer the duration of treatment [8, 9]. CTLA4 Ig, the only T-cell co-stimulator approved against RA, is prescribed primarily for RA patients who display an inadequate response to anti-TNF therapy. However, due to its good safety profile, CTLA4 Ig is gaining traction as an alternative therapy [10, 11]. Indeed, RA patients with prior exposure to TNF inhibitors were recently shown to have a greater 1-year risk of hospitalized infections compared with patients exposed to CTLA4 Ig [12].

The effect of biologics on host immune responses during different infections may vary significantly depending on the pathogens involved and the locations of infections. A detailed subgroup analysis for a specific infection is more important for clinical praxis than analysis of generalized infection risk. However, studies regarding the risk of *S*. *aureus* infections in RA patients treated with biologics are not very comprehensive. Recently, we demonstrated that both anti-TNF and CLTA4 Ig pre-treatment aggravated *S*. *aureus* systemic infection with different clinical manifestations in a mouse model of *S*. *aureus* septic arthritis [13]. The common route for *S*. *aureus* to initiate an invasive disease is through skin or mucosal colonization, skin barrier disruption, and accessing the adjoining tissues and blood stream [14]. To date, there is no clear clinical evidence suggesting that TNF inhibitors increase risk for skin and soft tissue infections [15, 16].

In this study, we investigated the effect of pretreatment with anti-TNF versus CTLA4 Ig on disease severity and bacterial clearance in a model of *S*. *aureus* skin infection. Our data demonstrate that both anti-TNF and CTLA4 Ig treatment attenuate the severity of *S*. *aureus* skin infection without affecting host bacterial clearance.

## Materials and methods

### Mice and animal experiment ethics statement

Female NMRI mice, 6–12 weeks old (purchased from Charles River Laboratories, Sulzfeld, Germany), were housed in the animal facility of the Department of Rheumatology and Inflammation Research, University of Gothenburg. Mice were kept under standard environmental conditions of temperature and light and were fed laboratory chow and water *ad libitum*. Experiments were approved by the Animal Research Ethical Committee of Gothenburg, and animal experimentation guidelines were strictly followed.

### Treatment with TNF-α inhibitor and CTLA4 Ig

Etanercept (Enbrel®; Wyeth Europa) was used for anti-TNF treatment because it fully inhibits biological function of murine TNF [17]. Abatacept (Orencia®; Bristo-Myers Squibb), a fusion protein of CTLA4 Ig, was used to modulate the co-stimulation of T-cells in mice [18]. Mice were administered either etanercept (5 μg/g of body weight) or abatacept (0.25 mg/g of body weight) in 0.1 ml of PBS via intraperitoneal injection (i.p.) twice a week starting one week before subcutaneous inoculation with *S*. *aureus* and continuing until sacrifice.

### Experimental protocols for staphylococcal skin infection

*S*. *aureus* SH1000 strains were prepared for infection experiments as previously described [19]. Briefly, pre-made aliquots of bacteria were thawed, washed and diluted to the desired concentration.

Six *in vivo* experiments were performed (S1 Fig). For all experiments, mice were divided into three groups (CTLA4 Ig, anti-TNF and PBS treatment groups). In experiments 1, 2, 3, and 5, the animals were anaesthetized with ketamine/medetomidine, their backs were shaved, and then they were injected subcutaneously (s.c) with 50 μl SH1000 suspension. Then, the resulting skin lesions were measured until the mice were sacrificed. All mice were regularly weighed, and skin lesions were measured with calipers. The sizes of the skin lesions were calculated using the mathematical formula for the area of an ellipse. The presence of dermonecrosis was defined as the appearance of brown on the skin surface, and/or a sunken area of the skin, and/or a red area of deepithelialization. Open skin lesions were defined as wounds on the skin accompanied by the complete loss of the epidermis and, occasionally, portions of the dermis or subcutaneous tissues. Two observers (M.N. and E.J.) who were blinded to the treatment groups inspected the lesion size of each mouse. At the end of experiments, the mice were anaesthetized with ketamine hydrochloride (Pfizer AB, Sweden) and metedomidine (Orion Pharma, Finland) before materials (blood, skin biopsies, and kidneys) was collected. Afterwards the mice were immediately sacrificed by a cervical dislocation.

The first experiment was performed to investigate the events following infection with high doses of bacteria. Twenty mice (6–7 mice/group) were injected s.c. with a relatively high dose of SH1000 (4.65×10^7^ CFU/spot). Changes to the skin were followed for 18 days, and the appearance of necrosis and wound healing was checked daily.

Twenty mice (6–7 mice/group) were used in the second experiment to study the histological changes and cytokine levels among groups. Mice injected s.c. with a relatively high dose of SH1000 (5.36×10^7^ CFU/spot) were sacrificed on day 6. Serum was sampled for cytokine analysis, and skins were collected for later histopathological examination. Because both experiments had similar outcomes, the results for skin lesion size were pooled with the results obtained from experiment 1.

The third experiment was performed to study the effect of a TNF inhibitor and CTLA4 Ig therapy on staphylococcal skin infection caused by a low bacterial dose. All mice (n = 7/group) were inoculated with a dose of 1.0x10^6^ CFU/spot on both flanks. After sacrificing the mice on day 4, the skin was collected for bacteriologic examination.

In experiment 4, 15 mice (n = 5/group) were anesthetized and injected intradermally (i.d.) into one ear pinna with 10 μl of SH1000 suspension (1x10^7^ CFU/spot), while the opposite side received the same volume of PBS as an internal control. The ear tissues were then collected for immune cell phenotyping.

The fifth experiment was designed to study the effect of a TNF inhibitor and CTLA4 Ig therapy on necrosis/apoptosis and on local cytokine levels in skin infection. All mice (n = 5/group) were injected i.d. into one ear pinna with 10 μl of SH1000 suspension (5x10^7^ CFU/spot), and the opposite side received the same volume of PBS as a control. Additionally, all mice were inoculated with a dose of 1.0x10^6^ CFU/spot on both flanks. After sacrificing the mice on day 2, the ear tissues were collected for apoptosis/necrosis detection, and the infected flank skin was collected and homogenized for cytokine analysis.

In experiment 6, we used a mouse model of superficial skin infections using a tape stripping method. The NMRI mice (n = 6-7/group) were anesthetized and had their backs shaved. Then, the skin on their flanks was stripped with an elastic adhesive bandage 7 times. An area of approx. 2 cm^2^ was stripped on each flank. Following this procedure, the skin was visibly damaged and was characterized by reddening and glistening but no frank bleeding. After stripping the skin, a bacterial infection was initiated by placing a superficial 5-μL droplet containing 2x10^7^ *S*. *aureus* SH1000. Lesion size and the wound healing time were then followed for 12 days.

### Skin homogenate preparation and bacteriologic examination

At the end of experiment 3, mice were euthanized, their skin was disinfected with 70% ethanol, and skin biopsies encompassing the entire infected area were taken with a sterile 8-mm biopsy punch (Kai Medical, Seki, Japan). Two biopsies from the injection sites on each flank were pooled and homogenized with an Ultra Turrax T25 homogenizer (IKA, Staufen, Germany), and then counts of viable bacteria in the homogenates were performed. This method recovers approximately 85% of the bacteria present in a skin sample[20]. At the end of experiment 5, the skin biopsies were collected and homogenized in 0.3 ml PBS as above. Thereafter, the homogenates were centrifuged at 13000 rpm for 10 minutes, and the supernatants were collected for cytokine analysis.

### Histopathological analysis

Skin biopsy samples were collected from mice infected with high doses of bacteria at day 6, fixed with 4% phosphate-buffered formaldehyde, embedded in paraffin, sectioned and stained with hematoxylin and eosin for histopathological evaluation. All slides were coded and assessed in a blinded manner by a pathologist (W.W). The widths and depths of lesions were measured. The degrees of bacteria load were scored on a subjective scale from 0 to 3. The presence of tissue necrosis, epithelial hyperplasia, and granulation were documented.

### Measurement of cytokine levels

The cytokine levels in serum were determined using the Cytometric Bead Array (CBA) Mouse Inflammation Cytokine Kit (BD Biosciences) and analyzed using the FacsCanto2 flow cytometer. The levels of IL-6, monocyte chemoattractant protein-1 (MCP-1), and interferon-gamma (IFN-γ) in the skin homogenates were determined using a DuoSet ELISA Development Kit (R&D Systems Europe, Ltd).

### Immune cell phenotyping of skin tissue

A total of 1x10^7^ CFU *S*. *aureus* SH1000 in 10 μl PBS were injected i.d. into the ear pinna of anesthetized mice (n = 5). On day 2 after infection, the ears were subjected to enzymatic digestion by 2 mg/ml Collagenase IV (Sigma-Aldrich) in PBS for 1 h at 37°C with shaking at 1400 rpm. After digestion, the samples were filtered with a 40-μm cell strainer (BD), washed with PBS, and stained with the indicated antibodies. The following antibodies were used: anti-mouse CD45-PE, anti-mouse CD11b-v450, anti-mouse Ly6G-APC, anti-mouse Ly6C-APC-Cy7 and anti-mouse F4/80-FITC (BD). Cell samples were analyzed with the FACSVerse flow cytometer (BD), and the data were analyzed with FlowJo (version 9.3.2; Three Star Inc., Ashland, USA).

### Detection of apoptosis/necrosis in skin tissue

Mice (n = 5/group) were injected i.d. into one ear pinna with 10 μl of SH1000 suspension (5x10^7^ CFU/spot), while the opposite side received the same volume of PBS as a control. On day 2 after infection, cell suspensions from the ears were obtained by enzymatic digestion as described above. Apoptosis/necrosis was detected using an apoptosis/necrosis detection kit (ab176750, abcam, Cambridge, UK). Cell samples were analyzed with the FACSVerse flow cytometer (BD), and the data were analyzed with FlowJo (version 9.3.2; Three Star Inc., Ashland, USA).

### Statistical analysis

Statistical significance was assessed using the Kruskal-Wallis test, Mann–Whitney *U* test, Fisher exact test and Mantel–Cox log-rank test. Results are reported as the mean ± standard error of the mean (SEM). A *p* value <0.05 was considered statistically significant. Calculations were performed using Prism v. 6.03 software (GraphPad Software, La Jolla, CA, USA).

## Results

### Both anti-TNF and CTLA4 Ig treatments reduced the skin lesion size caused by high doses of *S*. *aureus*

**Fig 1:**
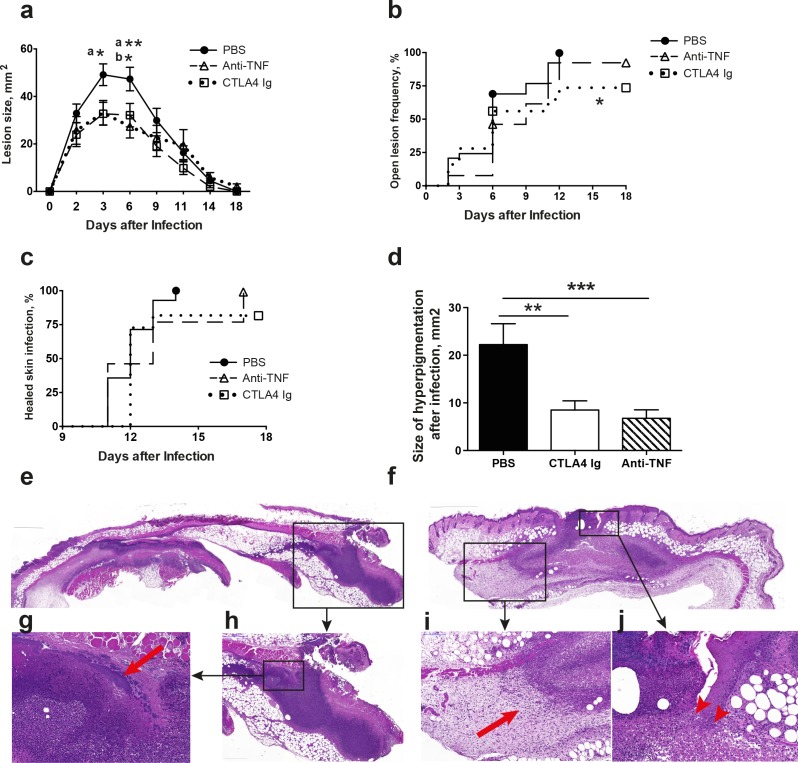
Both CTLA4-Ig and anti-TNF therapies attenuated the severity of *S*. *aureus* skin infection but tended to prolong healing time. NMRI mice (13-14/group) inoculated with a high dose of *Staphylococcus aureus* SH1000 (4.7–5.4×10^7^ colony-forming units/site) were treated with abatacept (CTLA4-Ig; 0.25 mg/g of body weight), etanercept (anti-TNF; 5 μg/g of body weight), or phosphate-buffered saline (PBS) twice weekly starting on day 7 before inoculation and continuing until the animals were euthanized on day 18. The lesion size (**a**), open lesion frequency (**b**), and wound healing time (**c**) of skin infection were observed for 17 days. (**d**) The size of skin hyperpigmentation on day 17 after infection. (**e-j**), Pathological changes in the skin of mice infected with *S*. *aureus* on day 6. Representative images of skin lesions from mice receiving PBS (**e**) or etanercept (**f**) are shown. (**g**) In PBS-treated mice, diffuse acute inflammation and necrosis are present in subcutaneous tissues, along with the presence of bacteria (arrowbar), but granulation formation is not present. (**f**) In etanercept-treated mice, the skin lesion is more limited, accompanied by evidence of granule formation (**i**, arrowhead) and epithelial hyperplasia (**j**, arrow). The data from two independent experiments were pooled. Statistical evaluations were performed using the Mann–Whitney *U* test, the Fisher exact test and the Mantel–Cox log-rank test. Data are the mean ± SEM. *P < .05, **P < .01, ***P < .001.

To determine the effects of anti-TNF and CTLA4 Ig treatment on the whole course of *S*. *aureus* skin infection in the subcutaneous injection model, the skin lesion size, frequency of open lesion, wound healing time, and size of skin hyperpigmentation were followed for 18 days after s.c. injection of a relatively high dose of *S*. *aureus* (Fig 1). At 2 days post-infection, the skin lesion became visible. From days 3–4, skin necrosis began to develop and a scar then formed. Starting on day 4, the necrotic tissue began to fall off and an open skin lesion appeared. Thereafter, the wound healing process began and a fully healed skin infection was first observed on day 11. Skin pigmentation was seen in most of the infection sites, with the exception of three mice receiving CTLA4 Ig and one receiving anti-TNF that never developed skin necrosis.

The size of the skin lesions increased during the early days after infection and peaked during day 3 to 6 (Fig 1A). Strikingly, anti-TNF treatment significantly reduced the skin lesion size on day 3 (p<0.05) and day 6 (p<0.01). Additionally, CTLA4 Ig treatment tended to attenuate skin damage on day 3 and had a significant effect at day 6 (p<0.05). Due to the wound healing process, the size of the skin damage decreased with time, and the differences among groups disappeared during the later stage of the disease.

The kidneys were collected at the end of experiments to evaluate abscess formation and bacterial burden in kidneys. Neither kidney abscesses nor bacterial growth from kidney homogenates was found, suggesting that anti-TNF and CTLA4 Ig do not facilitate the systemic spread of bacteria from a local skin infection.

Another skin infection model using the tape stripping method was also used to study the effects of the same treatments on the course of disease (S2 Fig). Here, the skin lesions were milder and the variation of skin lesion size was greater in comparison with the s.c injection method. No tangible difference regarding the skin lesion size was found among groups (S2A Fig). Interestingly, the wound healing time tended to be prolonged in the CTLA4 Ig group and significantly longer in mice treated with anti-TNF compared with the PBS group (S2B Fig).

### CTLA4 Ig treatment led to a prolonged course of disease

The open lesion forming after skin necrosis was common in mice inoculated with a high dose of *S*. *aureus* (Fig 1B). All mice administered PBS had an open skin lesion, whereas three mice receiving CTLA4 Ig did not develop skin necrosis or an open lesion until day 18 (100% vs 72%, p<0.05, Fig 1B). Of those three mice, two developed skin abscess instead. One mouse treated with anti-TNF never developed any sign of skin infections.

The wound healing process tended to be shorter in PBS control mice compared to those in the anti-TNF and CTLA4 Ig groups (Fig 1C). All open lesions in the PBS group were fully healed by day 14, whereas it took 17 days in the anti-TNF group. Of the CTLA4 Ig treated mice, two developed skin abscesses by the end of the experiment, which were deemed to be unhealed.

### Both anti-TNF and CTLA4 Ig treatments led to reduced skin hyperpigmentation after infection

Post-inflammatory hyperpigmentation following skin infections is a cosmetic problem in patients [21]. Interestingly, both anti-TNF (p<0.001) and CTLA4 Ig (p<0.01) treatments significantly decreased the size of skin hyperpigmentation on day 18 after skin infections (Fig 1D), strongly suggesting that both TNF-alpha and T-cell activation are responsible for post-inflammatory hyperpigmentation.

### Histological analysis of the effects of treatment on skin infections caused by high *S*. *aureus* doses

To confirm our clinical findings, a more detailed histologic analysis of skin infections was carried out on day 6 by a pathologist (W.W) who was blind to the treatment groups. In general, anti-TNF treatment led to more limited and less intense skin damage (Fig 1F) compared to PBS control mice (Fig 1E). Significantly narrower skin lesions (p<0.01, Table 1) were found in anti-TNF-treated mice compared with PBS-treated mice, whereas the depth of the lesions was similar across all groups. Importantly, epithelial hyperplasia (arrowhead in Fig 1J) was more common in the anti-TNF group than in PBS controls (50% versus 7%, p<0.05). Additionally, granulation (arrow bar in Fig 1I) was observed in 33% of mice receiving anti-TNF treatment compared with none of the PBS-treated mice (p<0.05). Our results from histological analysis confirmed our conclusions from clinical evaluation, namely that anti-TNF treatment attenuates skin damage in *S*. *aureus* skin infection.

**Table 1:** Histological analysis of skin biopsies from mice with *S*. *aureus* skin infections.

	Lesion size (mean)		Bacterial score	Necrosis	Epithelial hyperplasia	Inflammation		
	Depth,mm	Width, mm				Granulation	Acute	Chronic
**PBS (n = 13)**	1.7	7.10	2.0	100%	7%	0	100%	23%
**Anti-TNF (n = 12)**	1.9	5.65**	1.5	100%	50% *	33% *	100%	8%
**CTLA4-Ig (n = 14)**	1.8	6.85	1.5	100%	36%	7%	100%	21%

Statistical evaluations were performed using the Mann–Whitney *U* test and Fisher exact test. Data are presented as the mean value or frequency of events.

* = *p* < 0.05

** = *p* < 0.01.

### Anti-TNF treatment reduced the lesion size caused by a low dose of *S*. *aureus*

**Fig 2:**
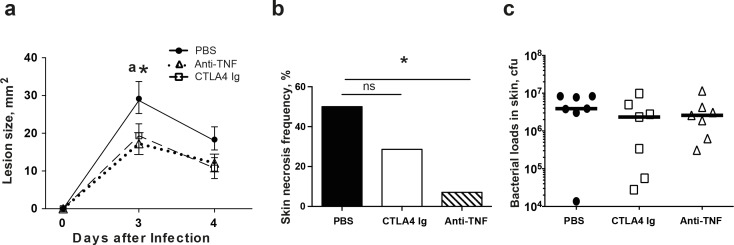
The attenuating effect of CTLA4 Ig and anti-TNF therapies on skin lesions was reproducible in milder *S*. *aureus* skin infection without affecting local bacterial load. NMRI mice inoculated with a low dose of *Staphylococcus aureus* SH1000 (1.0×10^6^ colony-forming units/site) were treated with abatacept (CTLA4-Ig; 0.25 mg/g of body weight; n = 7), etanercept (anti-TNF; 5 μg/g of body weight; n = 7), or phosphate-buffered saline (PBS; n = 7) twice weekly starting on day 7 before inoculation and continuing until the animals were euthanized on day 4. The skin lesion size (**a**) was observed for 4 days. The frequency of skin necrosis (**b**) and the bacterial load in skin (**c**) were analyzed on day 4 after infection. Statistical evaluations were performed using the Mann–Whitney *U* test and the Fisher exact test. Data are the mean ± SEM. *P < .05, ns = no significance. PBS, phosphate-buffered saline.

The clinical manifestations described above might differ in skin infections caused by different doses of *S*. *aureus*. Therefore, we carried out a similar experiment with a lower dose of *S*. *aureus* than before (Fig 2). Consistent with the data from the high dose experiment, significantly smaller lesions were observed on day 3 in the anti-TNF treatment group compared to PBS controls (p<0.05). However, the observed difference between the groups disappeared later in the experiment. CTLA4 Ig treatment also tended to decrease the lesion size; however, no significant difference was noted between CTLA4 Ig-treated mice and PBS controls. Importantly, on day 4, significantly more skin necrosis was found in PBS controls compared to mice treated with anti-TNF (50% versus 7.1%, p<0.05).

To study whether the decrease in skin damage is associated with an uncontrolled infection, kidneys and skin biopsies were collected and homogenized for bacterial counts. None of the mice had positive bacterial counts in the kidney homogenates. Neither anti-TNF nor CTLA4 Ig treatment had any impact on bacterial counts in locally infected skin (Fig 2C), strongly suggesting that neither treatment reduces the strength of the immune defense against bacterial proliferation.

### Serum and local skin cytokine profiles in mice with skin infection

**Fig 3:**
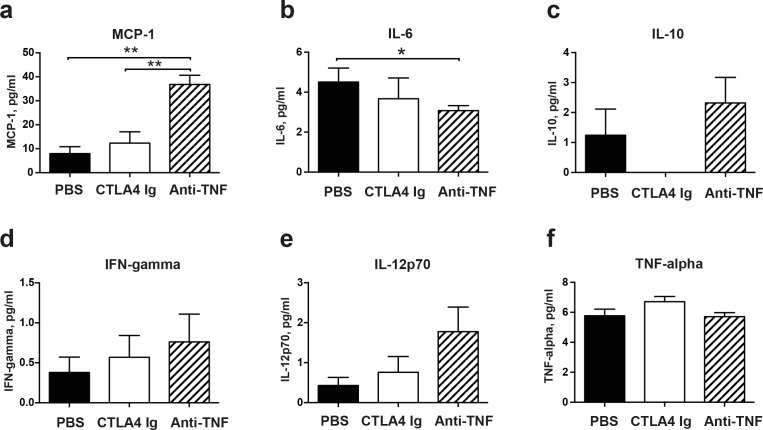
CTLA4 Ig and anti-TNF therapy resulted in different serum cytokine profiles of mice subcutaneously inoculated with *Staphylococcus aureus*. Serum levels of (**a**) monocyte chemoattractant protein-1 (MCP-1), (**b**) interleukin 4 (IL-4), (**c**) interleukin 6 (IL-6), (**d**) interleukin 10 (IL-10), (**e**) interferon γ (IFN-γ), (**f**) interleukin 12p70 (IL-12p70), and (**g**) tumor necrosis factor-alpha (TNF-α) were determined after termination of the experiment on day 6 after skin infection with a high dose of bacteria. Statistical evaluations were performed using the Kruskal-Wallis test and Mann–Whitney *U* test. Data are the mean ± SEM. *P < .05 and **P < .01. PBS, phosphate-buffered saline.

The serum levels of several cytokines and chemokines were analyzed on day 6 in mice infected with a high dose of *S*. *aureus* (Fig 3). The most striking difference was found in monocyte chemoattractant protein-1 (MCP-1) levels (Fig 3A): significantly higher MCP-1 levels were found in mice treated with anti-TNF than in both PBS controls (P<0.001) and CTLA4 Ig-treated mice (p<0.01). In contrast, IL-6 levels were lower in anti-TNF-treated mice than in PBS controls (p<0.05, Fig 3B). No significant difference was observed in the other cytokines analyzed, including IL-10, IFN-gamma, IL-12p70, and TNF-alpha.

**Fig 4:**
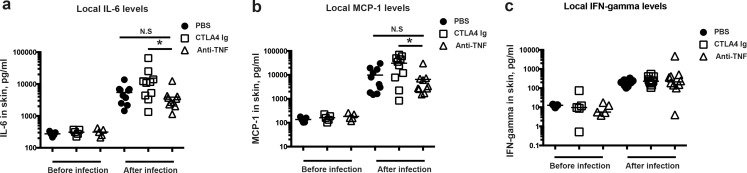
Skin tissue cytokine profiles of mice subcutaneously inoculated with *Staphylococcus aureus*. Levels of (**a**) interleukin 6 (IL-6), (**b**) monocyte chemoattractant protein-1 (MCP-1), and (**c**) interferon γ (IFN-γ) were determined in skin tissue homogenates after termination of the experiment on day 2 after skin infection with a low dose of bacteria (1.0x10^6^ CFU/spot). Statistical evaluations were performed using the Kruskal-Wallis test and Mann–Whitney *U* test. Data are the mean ± SEM. *P < .05 and **P < .01. PBS, phosphate-buffered saline.

The levels of MCP-1, IL-6, and IFN-γ in the healthy and infected skins were measured using ELISA kits (Fig 4). All cytokine levels in skin tissues were significantly elevated after infection. However, IL-6 and MCP-1 levels were not altered by anti-TNF or CTLA4 Ig treatment. Intriguingly, significantly lower IL-6 and MCP-1 levels were found in mice receiving anti-TNF compared with CTLA4 Ig-treated animals (*p*<0.05 for both). No tangible difference in tissue IFN-γ levels was found among groups.

### Anti-TNF treatment enhanced the number of locally infiltrating monocytes during skin infection

**Fig 5:**
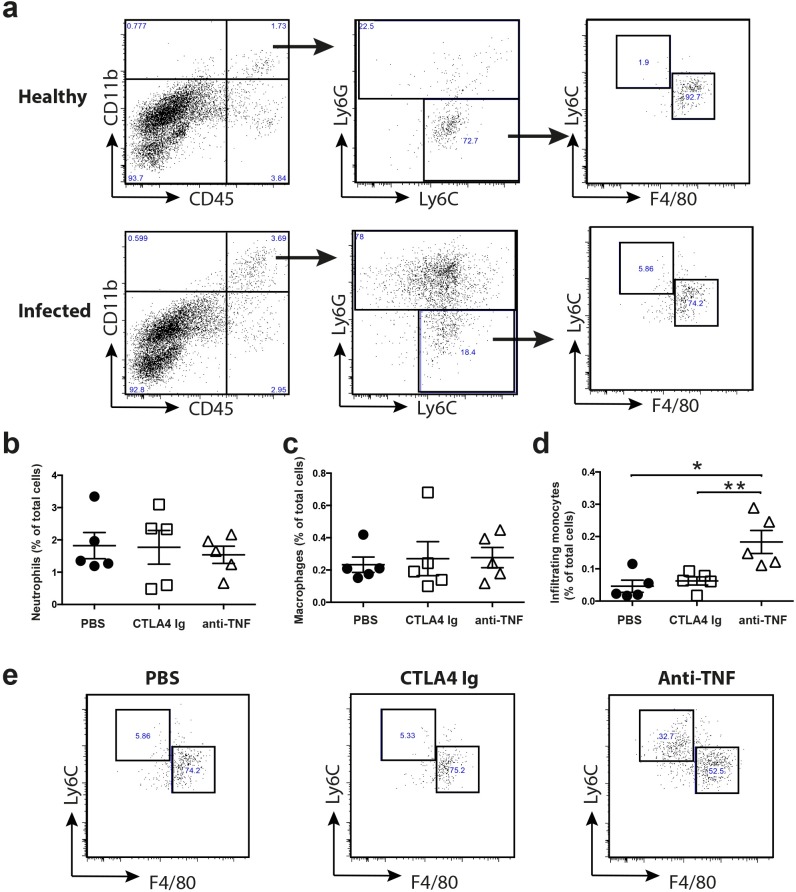
Anti-TNF treatment enhanced the number of locally infiltrating monocytes in *S*. *aureus* skin infection. (**a**) Representative FACS blots gated on CD45^high^CD11b^high^ cells before (0 h) and after (48 h) i.d. infection with 10^7^ CFU *S*. *aureus* SH1000. Proportions of (**b**) skin neutrophils, (**c**) macrophages, and (**d**) Ly6C^high^ inflammatory monocytes were analyzed in infected skin from mice treated with PBS, anti-TNF, or CTLA4 Ig. (**e**) Representative FACS blots gated on Ly6C^high^ F4/80^low^ in infected skin from mice treated with PBS, anti-TNF, or CTLA4 Ig. Statistical evaluations were performed using the Mann–Whitney *U* test. Data are the mean ± SEM. *P < .05 and **P < .01. PBS, phosphate-buffered saline.

Next, we studied whether infiltrating inflammatory cells in the skin were reduced in mice treated with anti-TNF and CTLA4 Ig. For this purpose, we analyzed the numbers of neutrophils, monocytes, and resident macrophages in the local sites of *S*. *aureus* skin infection (Fig 5). As expected, significantly increased number of neutrophils (CD11b+, CD45+, and Ly6G+ cells) and infiltrating monocytes were found in the skin at the infection site 2 days post-infection. Infiltrating monocytes were defined as CD11b++, CD45+, Ly6G-, and Ly6C^high^ cells. Large numbers of infiltrating monocytes were also found in the infected skin samples (Fig 5A). However, there were no differences in the numbers of infiltrating neutrophils or resident macrophages in infected skin samples between the treatment groups on day 2 post-infection (Fig 5B). To our surprise, the numbers of infiltrating monocytes in the infection sites were significantly higher in the anti-TNF group compared to both PBS controls (p<0.01) and the CTLA4 Ig group (p<0.05) (Fig 5B and 5C).

### Both anti-TNF and CTLA4 Ig treatments tended to down-regulate the necrosis/apoptosis ratio in the local skin

**Fig 6:**
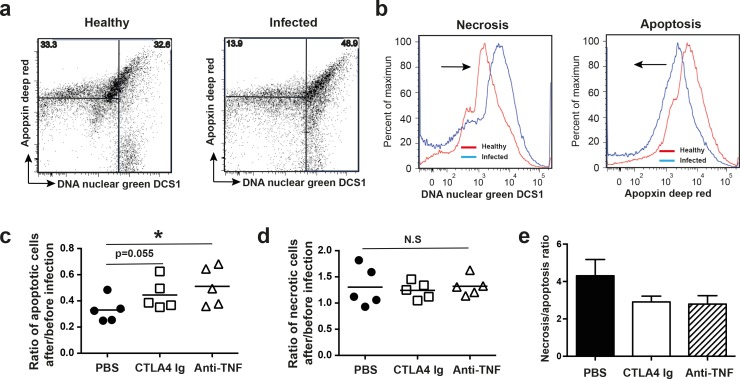
Both CTLA4 Ig and anti-TNF treatment tended to down-regulate the necrosis/apoptosis ratio in skin tissue. (**a**) Representative FACS blots gated on DNA Nuclear Green DCS1-positive (necrotic) cells and Apopxin Deep Red-positive (apoptotic) cells isolated from skin tissue infected i.d. with 5x10^7^ CFU *S*. *aureus* SH1000 in the ear pinna (right panel) and the opposing healthy ear (left panel). (**b**) Representative histogram of DNA Nuclear Green DCS1-positive (necrotic) cells and Apopxin Deep Red-positive (apoptotic) cells in healthy (red line) or infected skin (blue line). Ratio of (**c**) apoptotic cells and (**d**) necrotic cells in infected ear/healthy ear from mice treated with PBS, anti-TNF, or CTLA4 Ig. (**e**) Ratio of necrotic/apoptotic cells in the infected ears from mice treated with PBS, anti-TNF, or CTLA4 Ig. Statistical evaluations were performed using the Mann–Whitney *U* test. Data are the mean ± SEM. *P < .05 and **P < .01. PBS, phosphate-buffered saline.

To elucidate whether smaller skin lesions resulting from anti-TNF and CTLA4 Ig treatment were due to reduced necrosis, single cells prepared from healthy and infected ears were stained with DNA Nuclear Green DCS1 to indicate necrosis and Apopxin Deep Red for apoptosis (Fig 6). Fig 6A demonstrates typical necrosis/apoptosis FACS plots for cells from the uninfected ear (left panel) and the infected ear of the same mouse (right panel). The hallmarks of alterations in necrosis/apoptosis caused by infection in skin cells are significantly decreased numbers of apoptotic cells (Fig 6B, right panel) and increased necrotic cell counts (Fig 6A, left panel). Importantly, the mean ratio of apoptotic cells in the infected ear to the healthy ear was enhanced by anti-TNF treatment (0.33 vs. 0.51, p<0.05, Fig 6C) and CTLA4 Ig (0.33 vs. 0.44, *p* = 0.055). However, the ratio of necrotic cells in the infected ear to the healthy ear was not influenced by either of those treatments. The ratio of necrosis/apoptosis in the skin tended to be down-regulated by both CTLA4 Ig and anti-TNF treatment (Fig 6E).

## Discussion

In this study, we demonstrated that both anti-TNF therapy and CTLA4 Ig treatment significantly decreased the severity of skin infection and caused smaller post-infectious hyperpigmentation compared with controls. The beneficial effect was more pronounced with anti-TNF therapy than CTLA4 Ig. Importantly, none of the therapies administered had any effect on the capacity for bacterial clearance in the local skin. Neither anti-TNF nor CTLA4 Ig facilitated *S*. *aureus* skin infection to expand into a systemic infection. Our data suggest that in contrast to *S*. *aureus* systemic infection, *S*. *aureus* skin infection is not aggravated by anti-TNF therapy. It is important to note that mice receiving CTLA4 Ig had significantly fewer open skin lesions compared with PBS controls due to greater skin abscess formation instead of skin necrosis. Skin necrosis and subsequent drainage of suppurative lesions is one of the strategies that hosts use to eliminate infections and heal wounds [22]. Thus, it is not surprising that reduced skin necrosis caused by anti-TNF or CTLA4 Ig therapy tended to prolong wound healing in *S*. *aureus* skin infection.

We have previously shown that anti-TNF treatment deteriorates host bacterial clearance in a murine model for hematogenous *S*. *aureus* septic arthritis, resulting in more-severe weight loss and kidney abscesses [13, 17]. In contrast, in our present study, anti-TNF treatment decreased disease severity and caused smaller post-infectious hyperpigmentation without influencing bacterial burden in local tissues during staphylococcal skin infection. This strongly suggests a differential immune response between systemic infection and local skin infection to anti-TNF therapy. Tissue-necrotizing toxins and other virulence factors are known to dictate the severity of skin and soft-tissue infections caused by *S*. *aureus* [20, 23, 24]. Bacterial cell wall components (including peptidoglycan and teichoic acid) and enterotoxins directly contribute to the pro-inflammatory process, and TNF signaling is a crucial part of disease pathology in *S*. *aureus* infections [17, 25, 26]. TNF is capable of inducing skin tissue necrosis via TNF receptor 1 [27]. Local injection of a TNF antagonist in a periodontitis model results in diminished recruitment of inflammatory cells and periodontal destruction [28]. Tissue destruction in a gingivalis-induced skin abscess model is abolished in TNF-deficient mice [29], which is consistent with our results. Indeed, the reduction of the necrosis/apoptosis ratio in the local skin tissue by both anti-TNF and CTLA4 Ig treatment demonstrates the protective effect of those compounds against skin damage caused by *S*. *aureus* infection.

Neutrophils are considered to be the most potent effector cells during *S*. *aureus* skin infections [30]. Additionally, neutrophils are an important source of TNF-alpha in the mouse model of skin wounds [31]. Here, the number of infiltrating neutrophils was not affected by anti-TNF treatment, although a striking difference in skin lesion size was observed. During phagocytosis, neutrophils increase their oxygen consumption through the activity of an NADPH-oxidase that generates reactive oxygen species. Apart from killing microbes, the release of reactive oxygen species may also cause tissue damage [32]. TNF is known to act as a potent primer of neutrophil function via its receptor and phosphorylation of p47phox [33]. Therefore, blocking TNF may diminish the priming of neutrophils and subsequently reduce oxygen radical release and skin tissue damage.

Pre-treatment with CTLA4 Ig (abatacept) significantly increases the susceptibility of mice to *S*. *aureus* septic arthritis [13]. However, in the present study, we found that pre-treatment with CTLA4 Ig led to fewer skin lesions during local *S*. *aureus* skin infection. This is not quite unique because previous studies have demonstrated that blockade of T-cell activation by CTLA4 Ig prevents abscess formation in mice infected with different bacterial pathogens including *S*. *aureus* [34]. Recent results showed that T-cell signaling through CD28 contributes to MRSA pneumonia and that preventing T-cell co-stimulation significantly ameliorated the course of the disease in mice [35]. Additionally, staphylococcal enterotoxin-induced shock syndrome is prevented by CTLA4 Ig, with markedly reduced serum levels of TNF-alpha and IFN-gamma [36]. All of this evidence demonstrates the pathogenic roll of T-cell activation in certain *S*. *aureus* infections and the potential use of CTLA4 Ig to diminish tissue damage in those conditions.

Interestingly, serum MCP-1 levels were significantly elevated in anti-TNF-treated mice compared with mice in the CTLA4 Ig and PBS groups. MCP-1 is known as one of the key chemokines that regulate migration and infiltration of monocytes/macrophages. In accordance with this finding, the number of infiltrating monocytes in the locally infected skin was significantly increased in mice that received anti-TNF treatment compared with PBS controls. Previous work has demonstrated that infiltrating monocytes are rapidly recruited to the site of staphylococcal skin infection [37]. However, they have no apparent functional consequences (e.g., neutrophil recruitment, bacterial killing, or cytokine secretion) for the host response to *S*. *aureus* [37]. This phenomenon could help explain our current results whereby increased infiltration of monocytes seems to have no apparent effect on bacterial burden or on neutrophil recruitment to local infection.

One of the weaknesses of the current study includes the mode of inducing skin infection through subcutaneous injection of *S*. *aureus*. This method bypasses the first steps of a skin infection in clinical situations, i.e., skin colonization and subsequent invasion of *S*. *aureus* through the epidermis and dermis. Previous studies have shown that colonization with *S*. *aureus* significantly increases the risk of infections in patients in intensive care units [38, 39], while active surveillance together with subsequent decolonization decreases colonization by *S*. *aureus* and other hospital-acquired infections [40]. Additionally, in a multicenter study of bacteremia caused by *S*. *aureus*, the majority of cases of bacteria isolated in the bloodstream originated from colonies in the nasal mucosa [41]. Furthermore, a meta-analysis of published studies showed that colonization with methicillin-resistant *S*. *aureus* (MRSA) is associated with an increased risk of MRSA infection in dialysis patients [42]. Therefore, the question of whether anti-TNF treatment increases *S*. *aureus* skin colonization and consequently increases the risk of severe *S*. *aureus* infections has aroused significant interest. It has been suggested that treatment with anti-TNF agents alone is not associated with increased *S*. *aureus* carrier rates, whereas treatment with anti-TNF agents plus methotrexate in RA patients may predispose them to *S*. *aureus* colonization [43]. Interestingly, patients who are already colonized by *S*. *aureus* are more likely to remain colonized after anti-TNF therapy than patients who are not on biologics [44]. It seems that anti-TNF treatment slightly reinforces *S*. *aureus* skin colonization, which might increase the risk of a later skin infection. Although a more clinically relevant model (skin infection by abrasion) was used in this study, it remains unclear whether anti-TNF treatment or CTLA4 Ig has any impact on *S*. *aureus* invasion through the skin barrier. Therefore, the clinical relevance of our finding in mice needs to be further verified in patients receiving anti-TNF or CTLA4 Ig treatment.

Our data pinpoint the importance of TNF-alpha in mediating inflammation and tissue damage in staphylococcal skin infections. It is well known that tissue damage caused by *S*. *aureus* is not only a direct effect of the bacteria but also a result of exaggerated host response [17, 26, 45]. Proper antibiotics in combination with prednisolone therapy in patients with erysipelas shortened the healing time compared to patients receiving antibiotic alone [46]. It is unrealistic to use TNF inhibitor in patients with minor skin infections. However, preventing rapid tissue damage using a TNF inhibitor in combination with antibiotics might be an option for patients with much more severe diseases such as necrotizing fasciitis, a life-threatening infection involving the skin, soft tissue, and deep fascia. Actually, *S*. *aureus*, especially community-associated methicillin-resistant *S*. *aureus* (MRSA) is becoming more common as the cause of monomicrobial necrotizing fasciitis [47].

## Conclusion

In summary, our data suggest dual effects of CTLA4 Ig and anti-TNF therapies on *S*. *aureus* skin infection. On the one hand, both CTLA4 Ig and anti-TNF therapies attenuate the severity of *S*. *aureus* skin infections and have no impact on bacterial clearance in skin tissues. On the other hand, wound healing time was prolonged by both CTLA4 Ig and anti-TNF therapies, presumably due to reduced skin necrosis.

## Supporting information

S1 Fig Experimental protocol for *in vivo* experiments. (TIF)

S2 Fig Anti-TNF therapy prolonged the healing time of skin infection induced with a tape stripping method. NMRI mice (6-7/group) were treated with abatacept (CTLA4-Ig; 0.25 mg/g of body weight), etanercept (anti-TNF; 5 μg/g of body weight), or phosphate-buffered saline (PBS) twice weekly starting on day 7 before inoculation with bacteria and continuing until the animals were euthanized on day 12. Skin on mouse flanks was stripped with an elastic adhesive bandage, and a bacterial infection was initiated by placing a 5-μL droplet containing 2x10^7^ *S*. *aureus* SH1000 on the skin. The lesion size (**a**) and wound healing time (**b**) of skin infection in the mice were observed for 12 days. Statistical evaluations were performed using the Mann–Whitney *U* test and the Mantel–Cox log-rank test. Data are the mean ± SEM. *P < .05.
(TIF)
