# Supplementary figures and images for: Both anti-TNF and CTLA4 Ig treatments attenuate the disease severity of staphylococcal dermatitis in mice
Source: PLoS One. 2017 Mar 6;12(3):e0173492. doi: 10.1371/journal.pone.0173492 (PMC5338833; doi:10.1371/journal.pone.0173492)

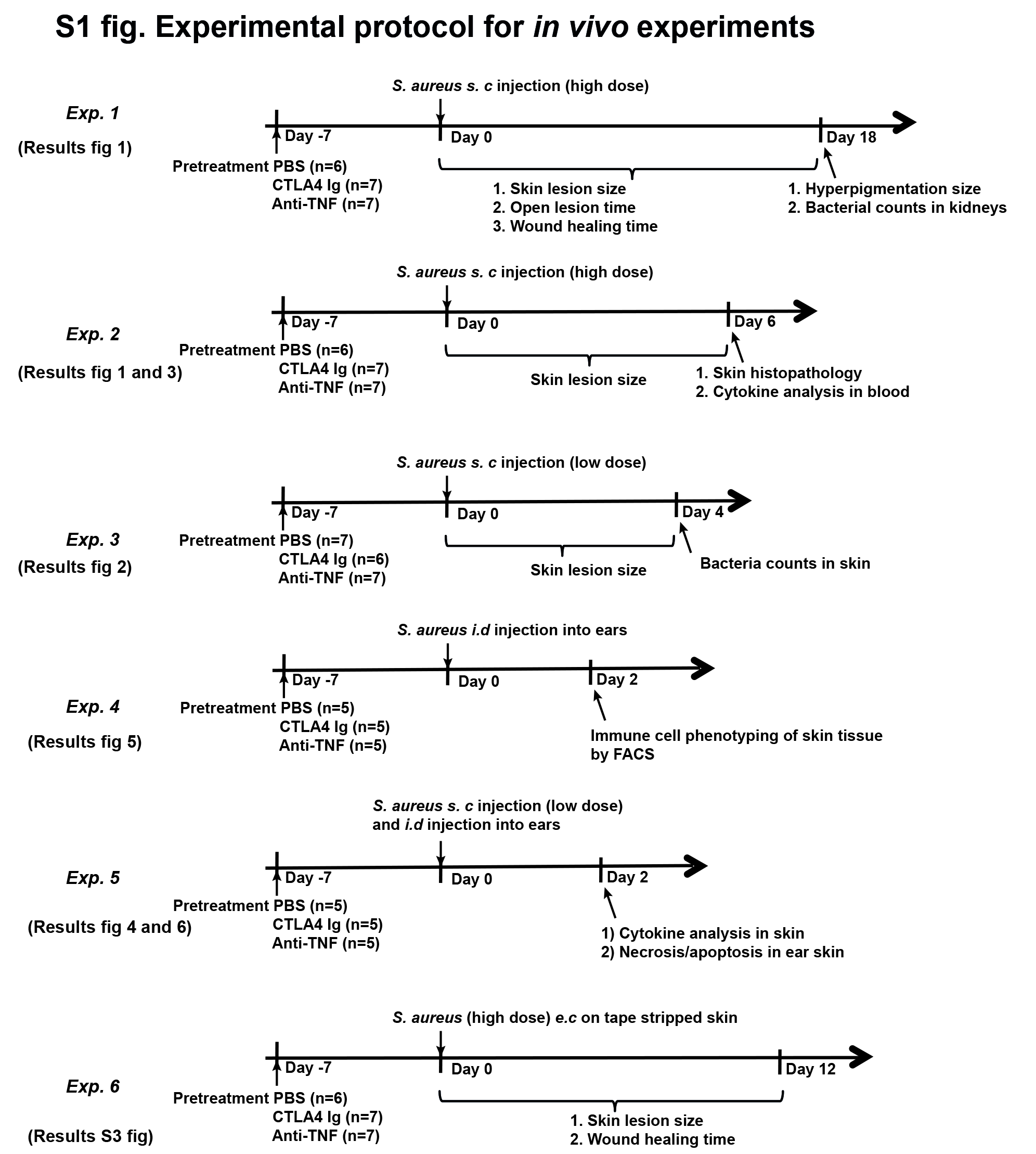

Supplement: S1 Fig — (TIF) [file pone.0173492.s001.tif]

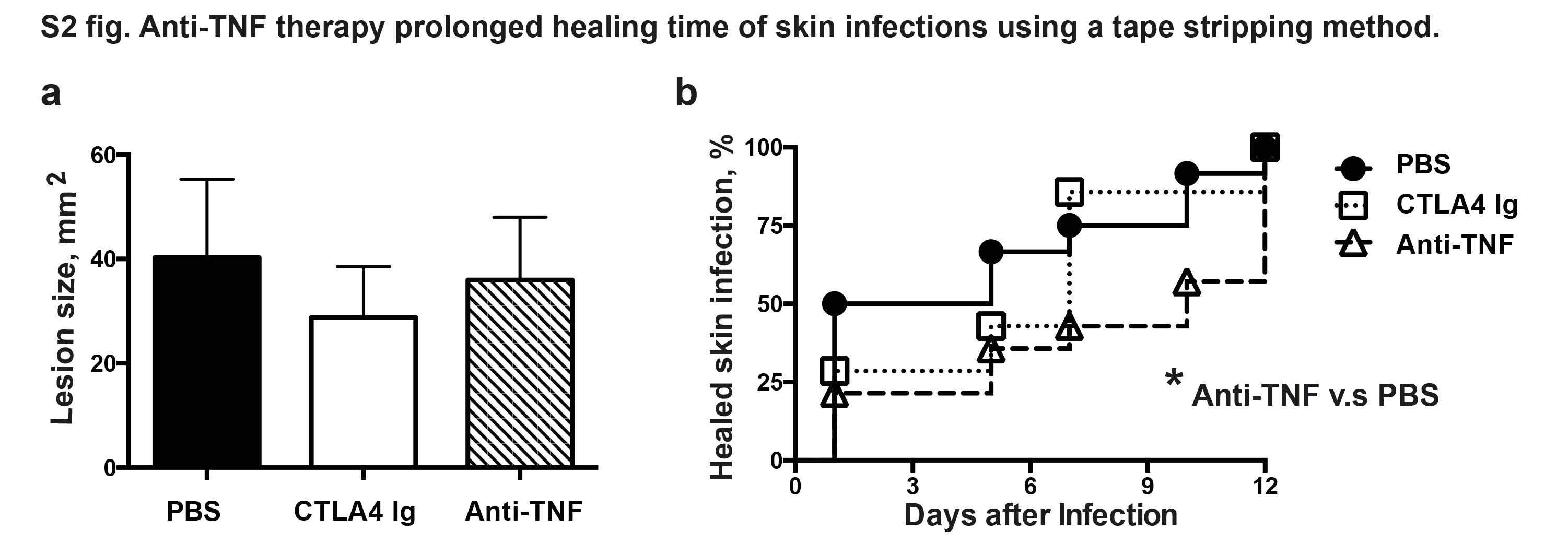

Supplement: S2 Fig — NMRI mice (6-7/group) were treated with abatacept (CTLA4-Ig; 0.25 mg/g of body weight), etanercept (anti-TNF; 5 μg/g of body weight), or phosphate-buffered saline (PBS) twice weekly starting on day 7 before inoculation with bacteria and continuing until the animals were euthanized on day 12. Skin on mouse flanks was stripped with an elastic adhesive bandage, and a bacterial infection was initiated by placing a 5-μL droplet containing 2x10^7^ S. aureus SH1000 on the skin. The lesion size (a) and wound healing time (b) of skin infection in the mice were observed for 12 days. Statistical evaluations were performed using the Mann–Whitney U test and the Mantel–Cox log-rank test. Data are the mean ± SEM. *P < .05. (TIF) [file pone.0173492.s002.tif]
